# Single cell analysis of Crohn’s disease patient-derived small intestinal organoids reveals disease activity-dependent modification of stem cell properties

**DOI:** 10.1007/s00535-018-1437-3

**Published:** 2018-01-27

**Authors:** Kohei Suzuki, Tatsuro Murano, Hiromichi Shimizu, Go Ito, Toru Nakata, Satoru Fujii, Fumiaki Ishibashi, Ami Kawamoto, Sho Anzai, Reiko Kuno, Konomi Kuwabara, Junichi Takahashi, Minami Hama, Sayaka Nagata, Yui Hiraguri, Kento Takenaka, Shiro Yui, Kiichiro Tsuchiya, Tetsuya Nakamura, Kazuo Ohtsuka, Mamoru Watanabe, Ryuichi Okamoto

**Affiliations:** 1 Department of Gastroenterology and Hepatology, Graduate School, Tokyo Medical and Dental University, Tokyo, 113-8519 Japan; 2 Department of Advanced Therapeutics in GI Diseases, Graduate School, Tokyo Medical and Dental University, Tokyo, 113-8519 Japan; 3 Center for Stem Cell and Regenerative Medicine, Graduate School, Tokyo Medical and Dental University, 1-5-45 Yushima, Bunkyo-ku, Tokyo, 113-8519 Japan

**Keywords:** Crohn’s disease, Intestinal stem cell, Intestinal organoids, Single cell analysis

## Abstract

**Background:**

Intestinal stem cells (ISCs) play indispensable roles in the maintenance of homeostasis, and also in the regeneration of the damaged intestinal epithelia. However, whether the inflammatory environment of Crohn’s disease (CD) affects properties of resident small intestinal stem cells remain uncertain.

**Methods:**

CD patient-derived small intestinal organoids were established from enteroscopic biopsy specimens taken from active lesions (aCD-SIO), or from mucosa under remission (rCD-SIO). Expression of ISC-marker genes in those organoids was examined by immunohistochemistry, and also by microfluid-based single-cell multiplex gene expression analysis. The ISC-specific function of organoid cells was evaluated using a single-cell organoid reformation assay.

**Results:**

ISC-marker genes, OLFM4 and SLC12A2, were expressed by an increased number of small intestinal epithelial cells in the active lesion of CD. aCD-SIOs, rCD-SIOs or those of non-IBD controls (NI-SIOs) were successfully established from 9 patients. Immunohistochemistry showed a comparable level of OLFM4 and SLC12A2 expression in all organoids. Single-cell gene expression data of 12 ISC-markers were acquired from a total of 1215 cells. t-distributed stochastic neighbor embedding analysis identified clusters of candidate ISCs, and also revealed a distinct expression pattern of SMOC2 and LGR5 in ISC-cluster classified cells derived from aCD-SIOs. Single-cell organoid reformation assays showed significantly higher reformation efficiency by the cells of the aCD-SIOs compared with that of cells from NI-SIOs.

**Conclusions:**

aCD-SIOs harbor ISCs with modified marker expression profiles, and also with high organoid reformation ability. Results suggest modification of small intestinal stem cell properties by unidentified factors in the inflammatory environment of CD.

**Electronic supplementary material:**

The online version of this article (10.1007/s00535-018-1437-3) contains supplementary material, which is available to authorized users.

## Introduction

The intestinal epithelium is a multifunctional tissue that helps maintain homeostasis and forms a physical barrier between the inside of the body and the outside world. Newborn intestine epithelial cells (IECs) arising from intestinal stem cells (ISCs) renew this epithelium every 4–5 days [1]. Deregulation of ISC- or lineage-specific functions is deeply involved in the pathogenesis of various gastrointestinal diseases, including inflammatory bowel disease (IBD) [2].

ISCs are found at the base of epithelial crypts [3]. Those crypt base columnar (CBC) cells are identified by series of ISC marker genes such as *LGR5*, *OLFM4*, *SMOC2* or *SLC12A2* [4, 5], and represent rapidly cycling ISCs. Also, another population of quiescent ISCs may exist at the ‘+ 4’ region of the crypts, which preferentially express genes such as *BMI1* or *HopX* [6, 7]. ISCs share the core stem cell properties, they self-renew and differentiate into five lineages of intestinal epithelial cells. Studies have shown that a heterogeneous group of cells share these ISC properties, and constitute a hierarchy within the ISC population [8]. A single-cell analysis clearly displayed this heterogeneity among the mouse small intestinal stem cells [9]. Other study has shown that LGR5^high^ cells located at the lowest part of the crypts divide rapidly and retain high stem cell activity, whereas LGR5^low^ cells residing at the + 4 region reduce their ability to initiate organoid culture [10]. Analysis of the human colonic crypt cells has also revealed the existence of distinct ISC sub-populations expressing *LGR5*, *ASCL2* or *OLFM4* [11]. However, the relevance of stem cell hierarchy or the heterogeneity to the pathophysiology of Crohn’s disease (CD) is poorly identified.

For the functional analysis of ISCs, the use of the intestinal organoid culture system has become a standard technique [12–14]. Organoids can be established from a single ISC in vitro [15], and faithfully retain the physiological and pathological features of their tissue of origin [16, 17]. Thus, organoids have been used to dissect underlying pathologic changes in various gastrointestinal disease [13, 18–20], in addition to the 2D-culture system [21]. A recent study identified permanent changes in gene expression patterns of colonic organoids established from ulcerative colitis (UC) patients [22], and indicated that colonic ISCs carry imprinted alterations possibly contributing to the perpetuation of the disease. However, whether such persistent or imprinted alterations exist in the small intestinal stem cells of CD patients remains unknown.

In our present study, we applied the single-cell analysis to CD patient-derived small intestinal organoids to identify modified intestinal stem cell properties. Using balloon-assisted enteroscopic biopsy samples, single-cell gene expression profiles of ISC-marker genes were identified in the organoids established from endoscopically active lesions, or from mucosa under remission and compared with those of non-IBD controls. Also, organoid reformation assay was performed to evaluate the potential stem cell function at the single-cell level. Through these studies, we aimed to clarify the possible influence of the inflammatory environment found in CD patients on the specific properties of small intestinal stem cells.

## Methods

### Collection of small intestinal tissues and ethical statements

Small intestinal enteroscopic biopsy samples were obtained from patients undergoing evaluation for diseases such as small intestinal tumors, occult bleeding, or Crohn’s disease. Up to 8 biopsies from each patient were taken from a region approximately 100 cm proximal to the ileocecal valve, during the evaluation for “endoscopic skipping” of the distal terminal ileum [23]. Also, surgical specimens of CD patients were collected to perform immunohistochemical analyses. Activity of CD was judged based on the endoscopic or macroscopic findings. The clinical backgrounds of patients are summarized in the Suppl. Table S1. The Ethics Committees of Tokyo Medical & Dental University and Yokohama Municipal Hospital approved our study (M2000-2093 and M2000-1176); and written informed consent forms were obtained from each patient.

### Immunohistochemistry and Immunocytochemistry

Immunohistochemistry and immunocytochemistry were done as previously described [24, 25]. The primary antibodies used are as follows: Rabbit-anti-human-OLFM4 mAb (1:1000, Cell Signalling Technology, Danvers, MA, USA); Mouse-anti-human-SLC12A2 Ab (1:1000, LS-Bio, Seattle, WA, USA); Mouse-anti-human-E-cadherin (1:500, Takara Bio, Kusatsu, Shiga, Japan). Signal amplification using fluorescence-conjugated Tyramide (Perkin-Elmer, Waltham, MA, USA) was employed for staining the proteins OLFM4 and SLC12A2.

### Crypt isolation and epithelial organoid culture

Isolation of the crypts and the subsequent establishment of intestinal organoids were performed as previously described [25, 26]. Briefly, crypts were collected by rigorously shaking biopsy specimens in 15 mM EDTA. Isolated crypts were embedded in 30 μl of Matrigel (BD Biosciences, Franklin Lakes, NJ, USA) at a density of 20–30 crypts per well and placed in 24-well culture dishes. The crypts were maintained in standard culture medium (WENR medium) consisting of basal medium (Advanced-DMEM, Thermo Fisher Scientific, Waltham, MA, USA) supplemented with recombinant human R-spondin-1 (1 μg/ml, R&D Systems, Minneapolis, MN, USA), recombinant human Wnt-3a (300 ng/ml, R&D Systems, Minneapolis, MN, USA), recombinant human Noggin (100 ng/ml, R&D Systems, Minneapolis, MN, USA), recombinant human EGF (50 ng/ml, PeproTech, Rocky Hill, NJ, USA), A83-01 (500 nM, Sigma-Aldrich Japan Tokyo, Japan) and SB202190 (10 μM, Sigma-Aldrich Japan, Tokyo, Japan). Y-27632 (10 μM, Sigma-Aldrich Japan, Tokyo, Japan) was added to the culture medium for the initial 3 days. Phase-contrast views of the established organoids were captured by a CCD-equipped microscope (BZ-X700, Keyence, Osaka, Japan).

### Single cell-level gene expression analysis

Cultured organoids were dissociated into single cells using TrypLE select (Thermo Fisher Scientific, Waltham, MA, USA) during the third culture passage. Single-cell suspensions were loaded onto the C1 preamp IFC (10–17 μm, Fluidigm San Francisco, CA, USA), for single-cell isolation. Single-cell capture was confirmed under a microscope (BX-X700, Keyence, Osaka, Japan). Pre-amplification of target genes was performed using TaqMan assays (Thermo Fisher Scientific, Waltham, MA, USA), with the IFC controller MX (Fluidigm, San Francisco, CA, USA). Further amplification and data acquirement of the multiplex quantitative PCR analysis was done using the Biomark 48.48. dynamic array IFC and Biomark HD system (Fluidigm, San Francisco, CA, USA), according to manufacturer’s instructions. The set of target genes, and the corresponding gene-specific TaqMan assays analyzed in the study can be found in the Suppl. Table S2.

## 3D scanner-based organoid re-formation analysis

After dissociating cultured organoids into single cells using TrypLE select (Thermo Fisher Scientific, Waltham, MA, USA), they were seeded onto 96-well plates at a density of 1 × 10^3^ – 1 × 10^5^ cells per well in 2 μl of Matrigel during the third culture passage. The cells were maintained in the WENR medium for 10 days. Organoid formation was quantified by scanning each well by a 3D scanner (Cell3 iMager, SCREEN Co., Kyoto, Japan) as previously described [25]. For the present study, we performed the organoid re-formation experiment and single-cell gene expression analysis at the same time for all groups side by side.

### Data processing and statistical analysis

Data acquired from multiplex single-cell gene expression analysis were processed using the Singular Analysis Toolset Software v3.5.2 (Fluidigm, San Francisco, CA, USA) and the Partek Genomic Suite (Version 6.6-6.16.0812, Partek, Chesterfield, MO, USA) by following standard workflows [27]. Statistical analysis was performed using the GraphPad Prism software (GraphPad Software, La Jolla, CA, USA), the XLSTAT (Addinsoft, New York, NY, USA) or the R statistics package.

## Results

### ISC-specific gene expression is altered in the active small intestinal lesions of CD patients

**Fig. 1:**
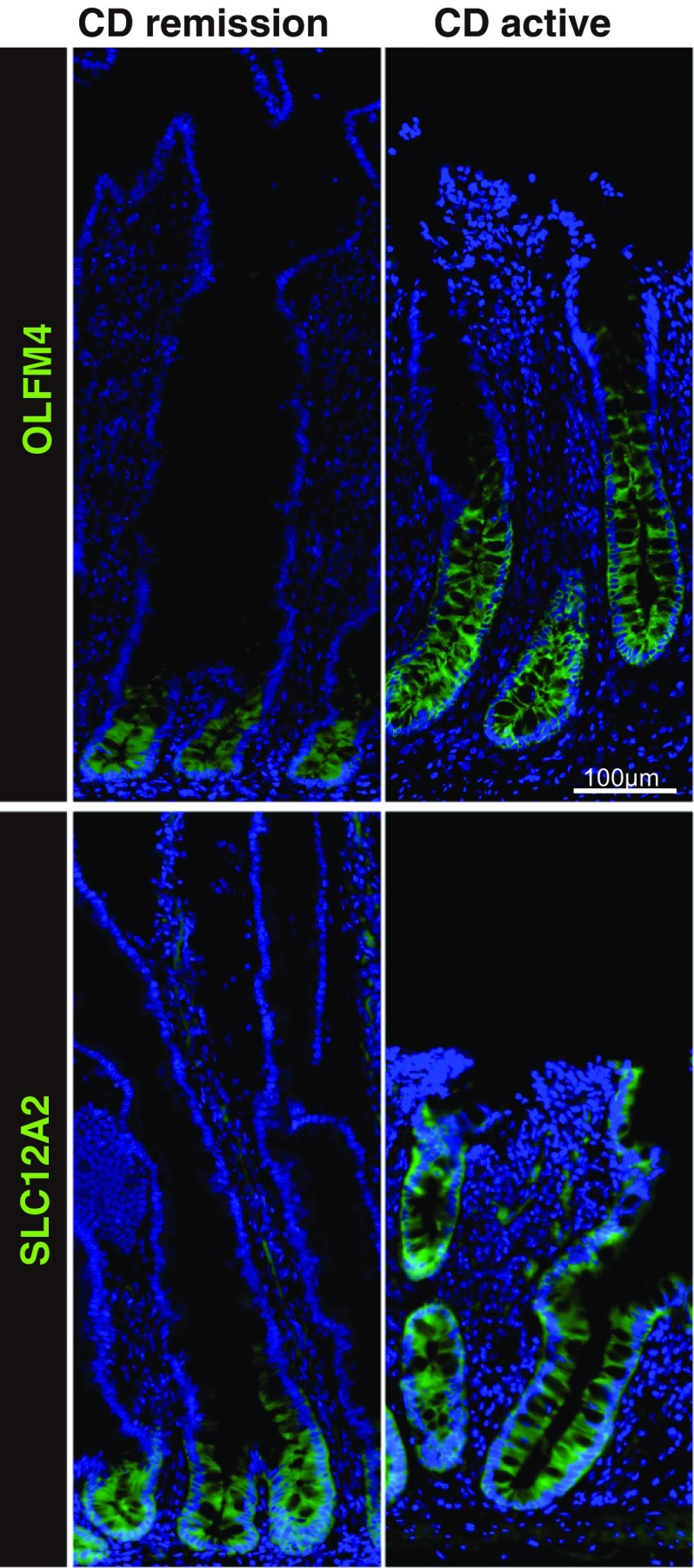
Immunostaining of small intestinal tissues for stem cell-specific genes, OLFM4 and SLC12A2. Small intestinal surgical specimens obtained from CD patients were subjected to immunostaining of OLFM4 and SLC12A2 (green). Results of small intestinal tissues obtained from active lesions of a CD patient (CD active) and those from CD patients in remission (CD remission) are shown. The same regions of the ileum were analyzed

To determine whether the small intestinal stem cell population is affected by the inflammatory environment in CD, we examined the expression of the ISC-specific genes, OLFM4 [28] and SLC12A2 [17, 29], by immunohistochemistry of surgical specimens. In the case of a CD patient in remission, OLFM4 and SLC12A2 were both expressed by intestinal epithelial cells (IECs) residing at the base of the crypt, including the crypt base columnar cells (Fig. 1). However, a relatively broad and intense staining of both OLFM4 and SLC12A2 was also observed in crypts at the active lesions of CD patients. Thus, indicating that the inflammatory environment of CD may affect the size of the small intestinal ISC population within the crypts. We also examined the staining of OLFM4 and SLC12A2 in endoscopic biopsy tissues and confirmed consistent difference between CD patients in active or in remission state of the disease (Suppl. Fig. S1).

### Small intestinal organoids can be efficiently established from CD patients

**Fig. 2:**
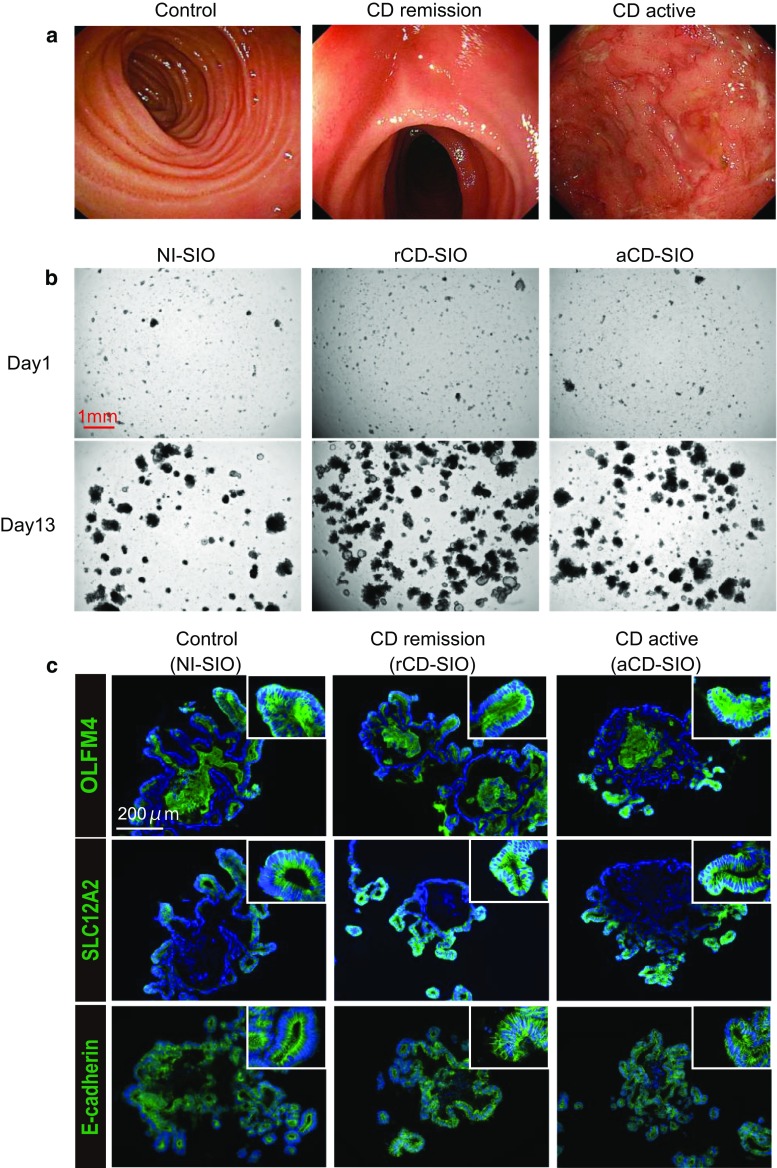
Establishment of small intestinal organoids from CD patients. **a** Enteroscopic view of the representative mucosa subjected to biopsy sampling: Control, patient #1; CD remission, patient #5; CD active, patient #7. **b** The efficient growth of small intestinal organoids in standard culture medium (WENR medium) can be seen. Data show representative phase-contrast views of patient-derived small intestinal organoids at passage 3: Control, patient #1 (non-IBD control, NI-SIO #1); CD remission, patient #4 (CD remission, rCD-SIO #4); CD active, patient #7 (CD active, aCD-SIO#7). **c** Immunostaining of SIOs at passage 3: Control, patient #2 (NI-SIO #2); CD remission, patient #4 (rCD-SIO #4); CD active, patient #7 (aCD-SIO #7). Immunostaining for OLFM4, SLC12A2 or E-cadherin (green) is shown. Positive signals in the organoid lumens may represent secreted forms OLFM4

To further characterize the small intestinal stem cell population in CD patients, we tested if patient-derived small intestine organoids could be established using enteroscopic small intestine biopsies taken from active lesions or from lesions under remission (Fig. 2a). Based on the methods described by Sato et al. [26], we confirmed the establishment of small intestinal organoids from both active lesions (aCD-SIO) and from lesions under remission (rCD-SIO, Fig. 2b and Suppl. Fig. S2). The crypts isolated from CD patients successfully generated organoids comparable to those of non-IBD controls (NI-SIOs). Immunostaining of those established organoids confirmed that they were solely composed of E-cadherin positive small intestinal IECs (Fig. 2c). Also, robust expressions of both OLFM4 and SLC12A2 at the budding lesion of the organoids allowed us to confirm recovery of patient-derived small intestinal stem cells (Fig. 2c and Suppl. Fig. S3). However, no clear difference in immunostaining patterns could be identified among organoids established from the three small intestinal organoid groups (Control, CD remission and CD active, Fig. 2c).

### Establishment and validation of single-cell analysis using CD patient-derived small intestinal organoids

**Fig. 3:**
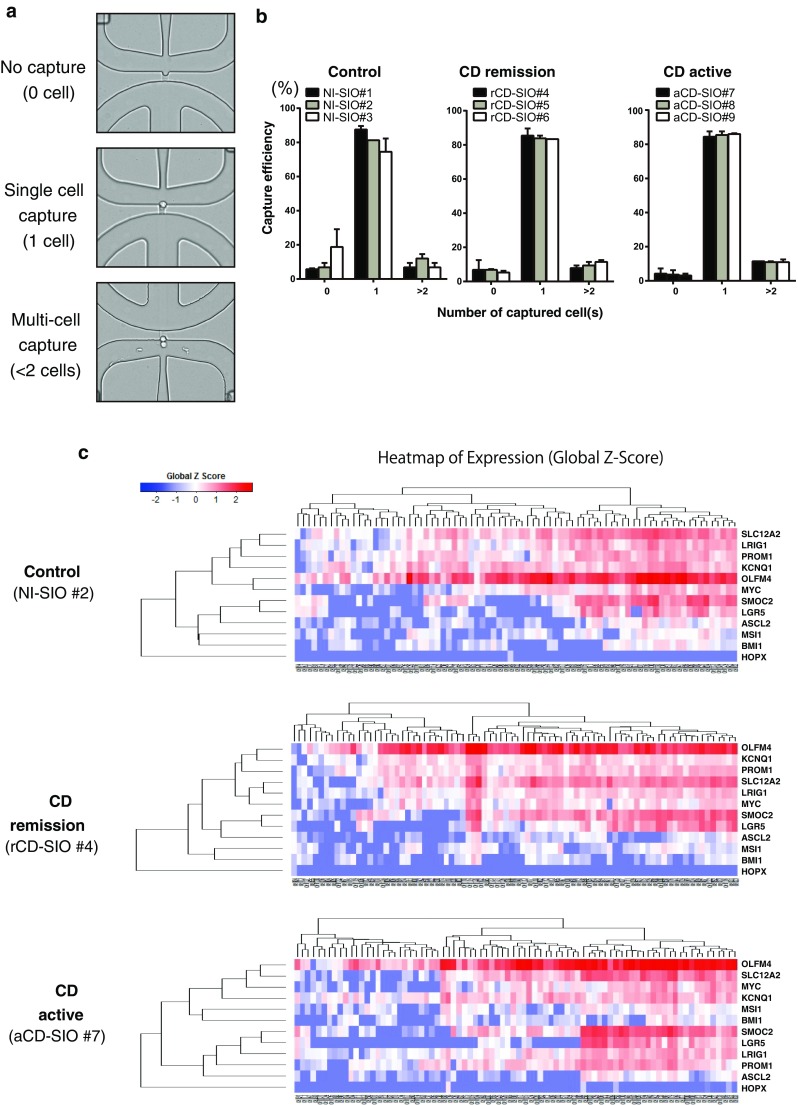
Establishment of single-cell analysis using patient-derived small intestinal organoids. **a** A representative picture showing successful and unsuccessful cases of a single-cell capture. **b** Efficiency of single-cell capture using patient-derived small intestinal organoids. Data are expressed as mean ± SEM of 2 rounds of capture. **c** Hierarchical analysis of single-cell data based on the expression profile of 12 intestinal stem cell (ISC)-specific genes. Single-cell gene expression data were acquired from small intestinal organoids established from non-IBD control (non-IBD control, NI-SIO #2), CD patient in remission (CD remission, rCD-SIO #4) or from an active lesion of a CD patient (CD active, aCD-SIO #7). 135 cells were subjected to the analysis for each small intestinal organoid. Gene expression profiles of individual cells after elimination of outlier cells are shown

To gain further insight into the gene expression pattern of CD patient-derived small intestinal stem cells we optimized our method for single-cell analysis [11]. We examined the cell-size distribution of the single-cell suspension, and found the diameter of dissociated human small intestinal epithelial cells reached more than 10 μm (the average diameter was 14.88 μm for organoid cells). Using the optimized microfluidic chip, we were able to capture a single intestinal organoid cell at around 80% efficiency (Fig. 3a, b). To identify the single-cell gene expression profile, over 100 small intestinal organoid-derived IECs were analyzed for each patient. We were able to obtain gene expression data for 12 ISC-marker genes (Suppl. Table S2). Hierarchical analysis of the data confirmed that the small intestinal organoids harbor a proportion of cells co-expressing multiple ISC-specific genes (Fig. 3c and Suppl. Fig. S4). Consistent with the immunostaining (Fig. 2c), both OLFM4 and SLC12A2 appeared to be expressed by a relatively wide range of cells. In contrast, LGR5 and SMOC2 were expressed by a relatively limited population of small intestinal IECs co-expressing multiple ISC-specific genes. Except for LRIG1, ISC markers representing ‘+ 4’ cells such as BMI1 and HopX were expressed at a low level indicating that those organoids are dominantly maintained by CBC-type ISCs. Also, LGR5 and SMOC2 showed similar expression patterns in all of the analyzed organoids, and were constantly classified at the closest distance within the gene hierarchy. Thus, our results suggest that the expression patterns of LGR5 and SMOC2 may be important for the hierarchical classification of small intestinal stem cells.

### Comprehensive analysis of ISC-marker gene expression in SIOs

**Fig. 4:**
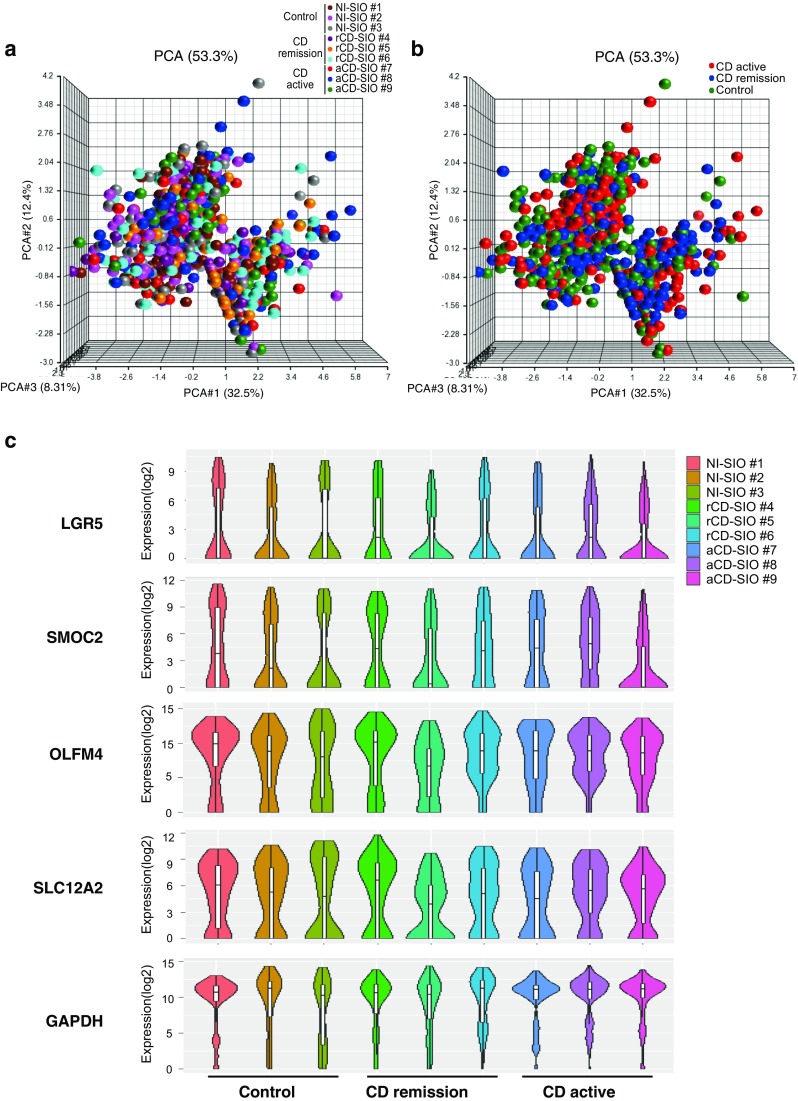
Limited variance of ISC-marker gene expression profile among cells constituting patient-derived small intestinal organoids. **a** Comprehensive principal component analysis (PCA) of the total single-cell gene expression data acquired from small intestinal organoids. Data shows distributions of gene expression variance at the single-cell level, after elimination of outlier cells (*n* = 1037). Color key indicates each patient-derived small intestinal organoid. **b** The same analysis shown in (**a**) displaying single-cell level gene expression variance of each small intestinal organoid group. **c** Violin plot showing single-cell gene expression level (*Y*-axis), and cell frequency (*X*-axis) in small intestinal organoids

**Fig. 5:**
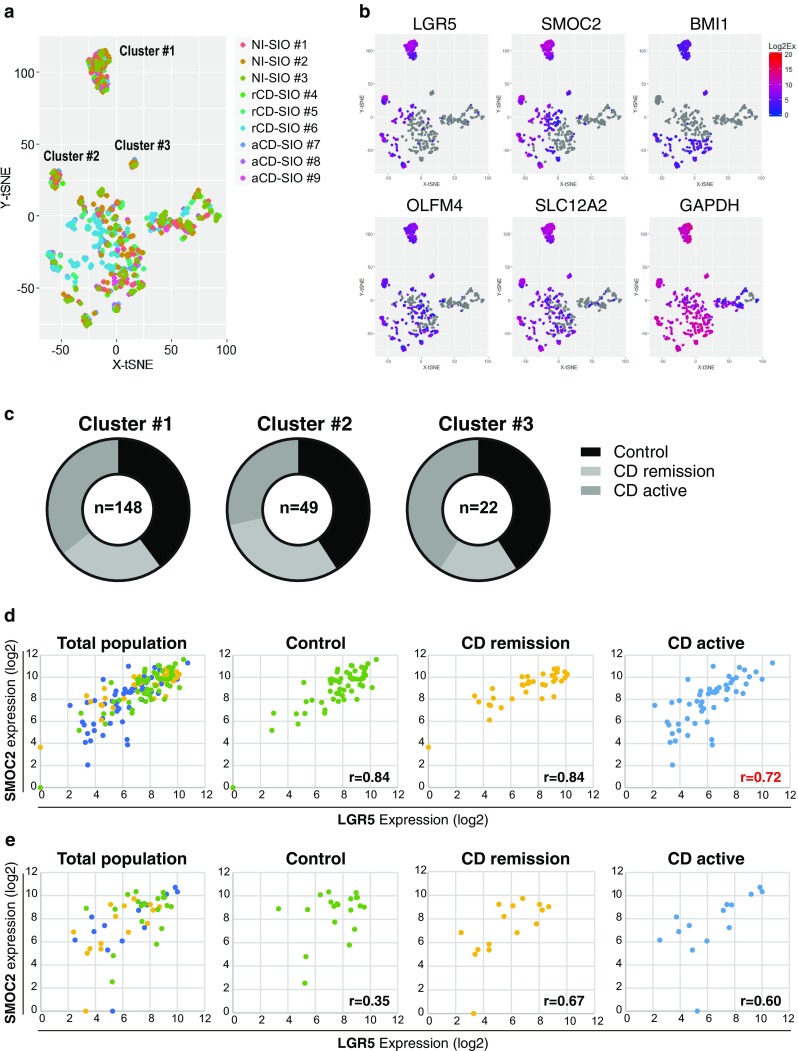
Identification of distinct cell clusters in small intestinal organoids based on single-cell level ISC-marker gene expression profile. **a** Data showing t-SNE analysis of the total single-cell gene expression data. Analysis was performed by the following conditions: perplexity = 17; iteration = 5000. Color key indicates each patient-derived small intestinal organoid. Note that cluster of cells is identified at the upper left region (Cluster #1), mid left region (Cluster #2) and also at the center region (Cluster #3). **b** Expression level of ISC-marker genes in individual cells. Redness intensity indicates the expression level of each gene. Gray color indicates negative expression. **c** Relative proportions of cells located in Cluster #1, Cluster #2 and Cluster #3. **d** Expression level of SMOC2 and LGR5 in individual cells located in Cluster #1. **e** Expression level of SMOC2 and LGR5 in individual cells located in Cluster #2

We next performed comprehensive analysis of the single-cell data. The principal component (PCA) analysis of the whole data revealed the possible existence of two major cell groups (Fig. 4a). However, the analysis did not clearly illustrate any patient specific, or study group specific distribution of the single-cell gene expression profiles (Fig. 4a, b). We compared the expression level of each gene among small intestinal organoids (Fig. 4c). The single-cell gene expression profiles for each ISC-marker gene were similar. Among the ISC-marker genes, LGR5 and SMOC2 showed a similar expression pattern that differed from those of OLFM4 and SLC12A2, confirming former observations of the hierarchical analysis. For further identification of small intestinal epithelial cell subgroups, we subjected our dataset to a t-distributed stochastic neighbor embedding (tSNE) analysis [30–33]. By analyzing the data based on similarity of cellular gene expression profiles, we identified three distinct clusters of small intestinal epithelial cells (Fig. 5a). In particular, both cluster #1 and cluster #2 were clearly conserved under different analysis conditions (Suppl. Fig. S5). Our results indicated that cluster #1 represents small intestinal epithelial cells co-expressing multiple ISC-markers at the highest level (Fig. 5b and Suppl. Fig. S6). Cluster #2 also represented a subgroup of small intestinal epithelial cells expressing several ISC-markers, but showing expression of BMI1 at an undetectable level. Cluster #3 was the smallest subgroup showing expression of LGR5 at an undetectable level. These cells expressed other ISC marker genes at a low level, and thus did not appear to be a good candidate for ISC sub-population.

The contents of these clusters sorted by their small intestinal organoid group showed comparable numbers of small intestinal epithelial cells from each group (Fig. 5c). Since we identified cluster #1 as the best candidate for small intestinal stem cell populations, we further analyzed if the ISC-markers were expressed by a study group-specific pattern within the intestinal epithelial cells of this cluster. The small intestinal epithelial cells of the control and the CD remission groups showed a highly correlated expression of SMOC2 and LGR5 in cluster #1 (Fig. 5d, Correlation coefficient: Control, *r* = 0.84; CD remission, *r* = 0.84). And, the small intestinal epithelial cells of the CD active group also showed a correlated expression in cluster #1, but with a lower correlation coefficient (*r* = 0.72). One-way multivariate analysis of variance (MANOVA) confirmed the distinct expression patterns of SMOC2 and LGR5 in the CD active group (Table 1). The correlated expression patterns of LGR5 and SMOC2 were not conserved in the epithelial cells of cluster #2, compared to those in cluster #1 of the corresponding organoid group (Fig. 5e). These results indicated that small intestinal organoids derived from active lesions of CD (aCD-SIOs) may contain a stem cell population that is distinct from that of the other two organoid groups.

**Table 1:** Results of one-way MANOVA comparing expression of LGR5 and SMOC2 in 3 groups of cluster#1 small intestinal epithelial cells

Compared groups	*F* value (critical value)	Significance
Control vs CD remission vs CD active	*F* (4, 288) = 5.277 (2.403)	*P = 0.000408
Control vs CD remission	*F* (2, 92) = 2.477 (3.095)	*P* = 0.089617
Control vs CD active	*F* (2, 109) = 9.736 (3.080)	*P = 0.000128
CD remission vs CD active	*F* (2, 86) = 4.551 (3.103)	*P = 0.013227

### Active CD organoids harbor increased numbers of organoid-reforming small intestinal stem cells

**Fig. 6:**
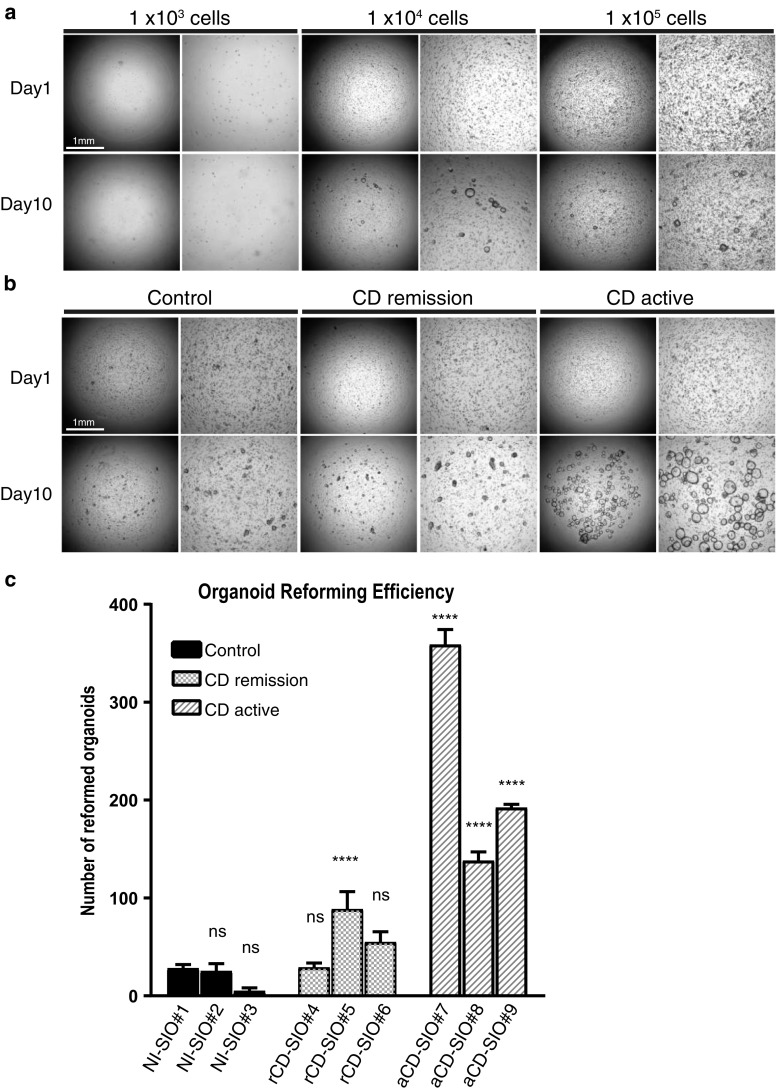
Active CD-small intestinal organoids harbor increased number of ISCs retaining organoid reformation ability. **a** Optimization of single-cell based organoid reformation assay. NI-SIO #2 was dissociated into single cells, and seeded onto a 96-well plate at the designated cell density. Data shows phase contrast view at the beginning of the culture and after 10 days of culture in WENR medium. **b** Single-cell based organoid reformation assay using patient-derived small intestinal organoids. Organoids were dissociated into single cells, and seeded onto 96-well plate at a density of 1 × 10^4^ cells per well. Data shows phase contrast view at the beginning of the culture and after 10 days of culture in WENR medium. **c** Quantification of organoid reformation efficiency for each small intestinal organoid. Data are shown as mean ± SEM of triplicate analysis. ****Indicates *P* < 0.0001 determined by two-way ANOVA, compared to NI-SIO#1

As our single-cell gene expression analysis indicated modified intestinal stem cell-marker gene expressions in active CD organoids, we sought to evaluate the ISC-specific functions of those stem cells. Reformation of organoids from single cells has been employed to verify the self-renewal capabilities of potential ISCs [15]. Therefore, we established a single cell-based organoid reformation assay, assisted by a newly developed 3D-scanner [25]. In this case, we first optimized the primary cell density and the evaluation period. As a result, we found that a seeding density of 1 × 10^4^ cells per well provides reliable and accurate quantification of organoid reformation after 10 days (Fig. 6a). We then compared the organoid reforming efficiency of each small intestinal organoid-derived epithelial cell; and found that those cells derived from remission organoids showed comparable reformation efficiency to those derived from non-IBD organoids (Fig. 6b, c). Although remission sample rCD-SIO#5 showed significantly higher organoid reformation efficiency compared to the non-IBD NI-SIO#1 sample. However, in sharp contrast, small intestinal epithelial cells derived from active CD-organoids showed higher reformation efficiencies, and were able to reform larger organoids, than those in other organoid groups (Fig. 6b, c). Thus, our results clearly indicate that active CD organoids harbor increased numbers of small intestinal epithelial cells that retain their ISC-specific organoid reconstruction function.

## Discussion

Through our present study we successfully established CD patient-derived small intestinal organoids from enteroscopic biopsies. Previous studies have reported that colonic organoids established from CD or UC patients show sustained alteration in gene expression patterns or differentiation potential [22]. Compared to the former study, we found limited alteration in comprehensive ISC-marker gene expression in CD patient-derived small intestinal organoids, compared to non-IBD patient-derived small intestinal organoids. Also, hierarchical analysis showed variance among patients, suggesting a certain range of disease-unrelated heterogeneity and also variance in the disease.

However, through our analysis of a total 1037 cells, we identified at least 3 clusters of small intestinal epithelial cells expressing ISC-markers (Fig. 5a). Those clusters could be clearly discriminated by the expression of LGR5 and BMI1. At present, it is not clear how these clusters correspond to the hierarchical population of small intestinal stem cells or progenitor cells in vivo. But, since the small intestinal epithelial cells of cluster #1 expressed multiple ISC-makers including LGR5 and SMOC2 at the highest levels, they may well correspond to the genuine crypt base columnar cells. In contrast, the epithelial cells of cluster #2 and cluster #3 showed expression of LGR5 or BMI1 at an undetectable level, suggesting that those cells may correspond to epithelial cells residing at positions higher than + 4, where the lineage-specific progenitors mainly reside [34, 35]. However, further functional and deeper gene expression studies would be needed to identify the in vivo relevance of these epithelial cell clusters.

In contrast to LGR5, BMI1 and SMOC2, high-level expression of OLFM4 and SLC12A2 were observed generally in a large population of cells, including those expressing LGR5, BMI1, and SMOC2 at a very low level (Fig. 3c and Suppl. Fig. S3). The broad expression of these genes may indicate that their expression level is maintained at a relatively high level in early progenitor cells, in addition to ISCs.

Our analysis also revealed a tight correlation between SMOC2 and LGR5 expression in the cells of cluster #1. Both markers have been identified as having expressions highly dependent on the intracellular Wnt pathway activity [4]. This finding may indicate one of the transcriptional identities of genuine ISCs. However, the significant disruption in this SMOC2–LGR5 correlation in the cluster #1 epithelial cells derived from active CD organoids may suggest that a yet unknown inflammatory factor is interfering.

In our present study, we found comparable primary organoid forming capacity for biopsies derived from inflamed tissue compared to biopsies derived from uninflamed tissue (Fig. 2b). Such a result is in clear contrast to the data acquired from organoid cells (Fig. 6b). The most important difference in these two data is the condition of cells at the beginning of culture. For the culture shown in Fig. 2b, dissociated organoid fragments were subjected to culture, thus representing the organoid forming potential per fragment. In contrast, for the culture shown in Fig. 6b, single isolated cells were subjected for culture, thus representing the organoid forming potential per cell. The clear difference in these two data may indicate that single cell-based culture is more appropriate to identify the size of potential stem cell population within an organoid.

Our functional analysis further revealed high organoid reformation efficiency of small intestine epithelial cells constituting active CD organoids. Such an observation indicates that increased number of potential ISCs are present in these organoids. It may be possible that active CD organoids carry increased numbers of epithelial cell progenitors that retain high plasticity to re-acquire properties of ISCs. Recent studies in mice have revealed the unexpectedly high plasticity of lineage-restricted IECs [7, 36]. Also, inflammatory signals mediated by the NF-kB pathway can revert differentiated intestinal epithelial cells back into ISCs in mice [37]. We have recently shown that colonic IECs of DSS colitis mice acquire increased ability to form organoids ex vivo, possibly by the recruitment of secretory-lineage cells to the ISC pool [38]. Therefore, the high organoid re-forming ability of CD-active group organoids may be supported both by genuine stem cells and inflammation-induced stem cells. Also, the distinct SMOC2–LGR5 expression patterns of cluster #1 epithelial cells in active CD organoids may be the result of increased heterogeneity by persistence of reprogrammed ISCs. It may be possible that adding further genes for analysis, or performing single-cell RNA-seq analysis, may better identify active CD specific cell population that has high organoid reforming ability.

Although we observed a clear functional difference in the small intestinal epithelial cells of active CD organoids, we cannot be certain that this was due to the unique inflammatory environment in vivo. But, it is conceivable that the high organoid reformation ability of the active CD organoid-derived epithelial cells is a feature contributing to the regeneration of damaged small intestine mucosa in vivo. Currently, it is not clear how long the organoids derived from actively inflamed intestinal mucosa can retain their inflammatory properties ex vivo. Previous study by Dotti et al. [22] reported that organoids established from UC patients show “permanent” phenotypes possibly induced by the host inflammatory environment. However, in their study, it was not clear whether the disease-related phenotypes depend on passage number or on culture period of patient-derived organoids. As we found a clear functional phenotype specific for active CD-derived organoids (Fig. 6), we could at least say that properties of actively inflamed intestinal mucosa may persist up to third culture passage. Further analysis of the small intestinal epithelial cell population responsible for such a high organoid reformation capacity may identify the specific cells needed to heal the mucosa in CD patients.

Also in our present study, we could not find any treatment-specific phenotype of patient-derived organoids. However, as for the limited number of patient-derived organoids included in the present study, it remains possible that analysis of larger number of patient-derived organoids may uncover treatment-specific phenotype among those organoids.

Small intestinal epithelial cells with high organoid reformation capacity may be useful in transplantation therapies. A previous study have shown that small intestinal organoids can engraft onto the damaged colonic epithelium of mice models, and reside there as a small intestinal epithelium [39]. Our active CD organoids may have a high regenerative potential that might be applied for the treatment of refractory small intestine ulcers [40, 41].

In summary, we developed a single-cell analysis system for small intestinal organoids and found modifications of ISC properties in active CD organoids. The present single-cell level system may be extended to analyze colonic organoids of CD or UC, to gain further insight into the pathophysiology of ISCs.

## Electronic supplementary material

Below is the link to the electronic supplementary material.

Supplementary material 1 (PDF 4994 kb)

Supplementary material 2 (TIFF 2425 kb)

Supplementary material 3 (PDF 108 kb)

Supplementary material 4 (PDF 1218 kb)

Supplementary material 5 (PDF 234 kb)

Supplementary material 6 (PDF 228 kb)

Supplementary material 7 (PDF 69 kb)

## Abbreviations

ISC: Intestinal stem cells
CBC: Crypt base columnar
CD: Crohn’s disease
UC: Ulcerative colitis
IBD: Inflammatory bowel disease
IEC: Intestinal epithelial cell
aCD-SIO: Active Crohn’s disease-derived small intestinal organoid
rCD-SIO: Small intestinal organoid of Crohn’s disease in remission
NI-SIO: Small intestinal organoid of non-IBD control
tSNE: t-distributed stochastic neighbor embedding
MANOVA: Multivariate analysis of variance
