# Supplementary material for: Single cell analysis of Crohn’s disease patient-derived small intestinal organoids reveals disease activity-dependent modification of stem cell properties
Source: J Gastroenterol. 2018 Jan 27;53(9):1035–47. doi: 10.1007/s00535-018-1437-3 (PMC6132922; doi:10.1007/s00535-018-1437-3)
Supplement: Supplementary file 1 — Supplementary material 1 (PDF 4994 kb) [file 535_2018_1437_MOESM1_ESM.pdf]

Supplementary Figure S1

a.

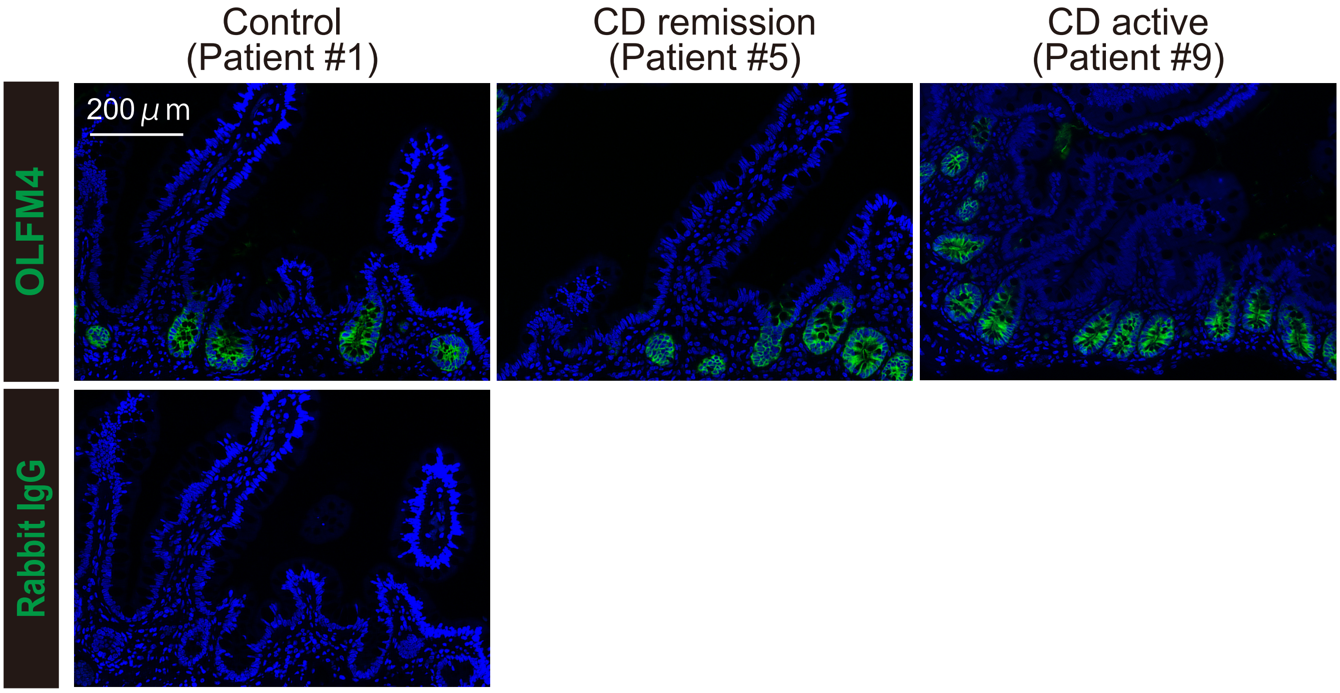

b.

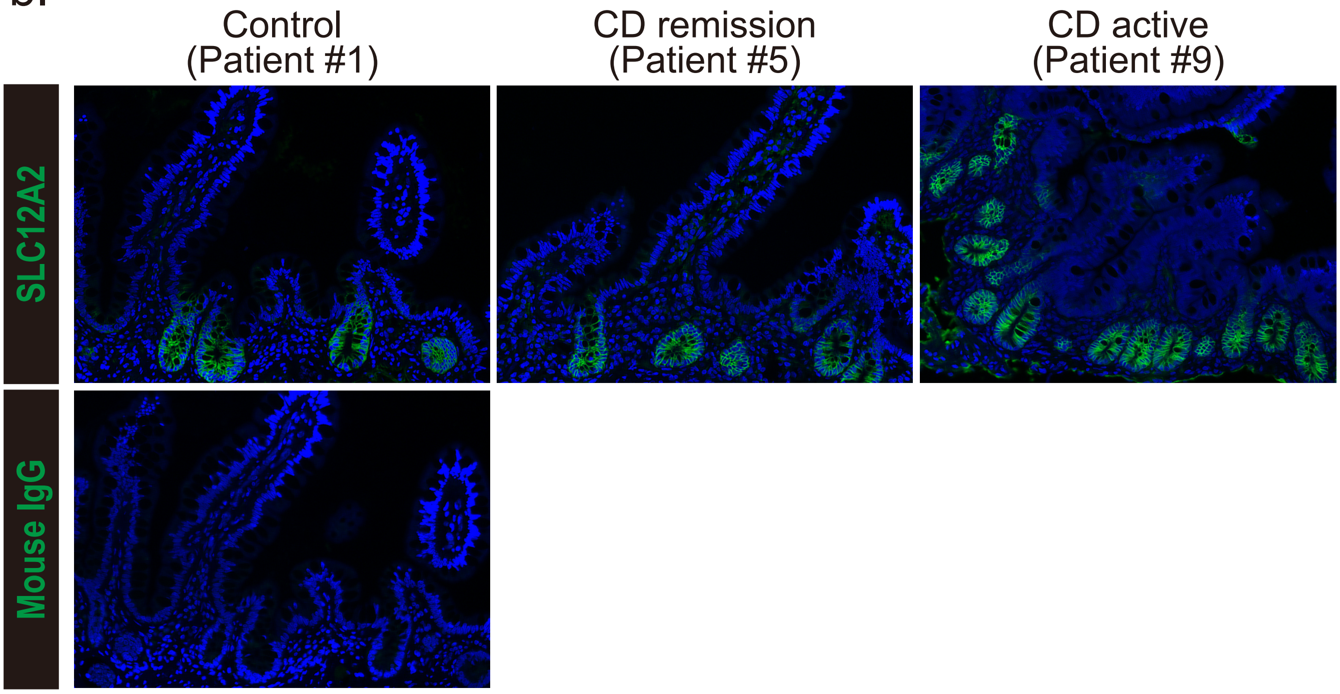

Supplementary Figure S1. Expression of OLFM4 and SLC12A2 in biopsy specimens. Expression of OLFM4 (a) and SLC12A2 (b) were analyzed by immunohistochemstry using small intestinal biopsies. Positive signals are shown as green signals of the fluorescein-labeled tyramid. Results of corresponding non-immunized control IgG are also shown. Data shows results of the same area (100 cm proximal to the ileocecal valve).
