# Supplementary material for: Single cell analysis of Crohn’s disease patient-derived small intestinal organoids reveals disease activity-dependent modification of stem cell properties
Source: J Gastroenterol. 2018 Jan 27;53(9):1035–47. doi: 10.1007/s00535-018-1437-3 (PMC6132922; doi:10.1007/s00535-018-1437-3)
Supplement: Supplementary file 3 — Supplementary material 3 (PDF 108 kb) [file 535_2018_1437_MOESM3_ESM.pdf]

Supplementary Figure S3

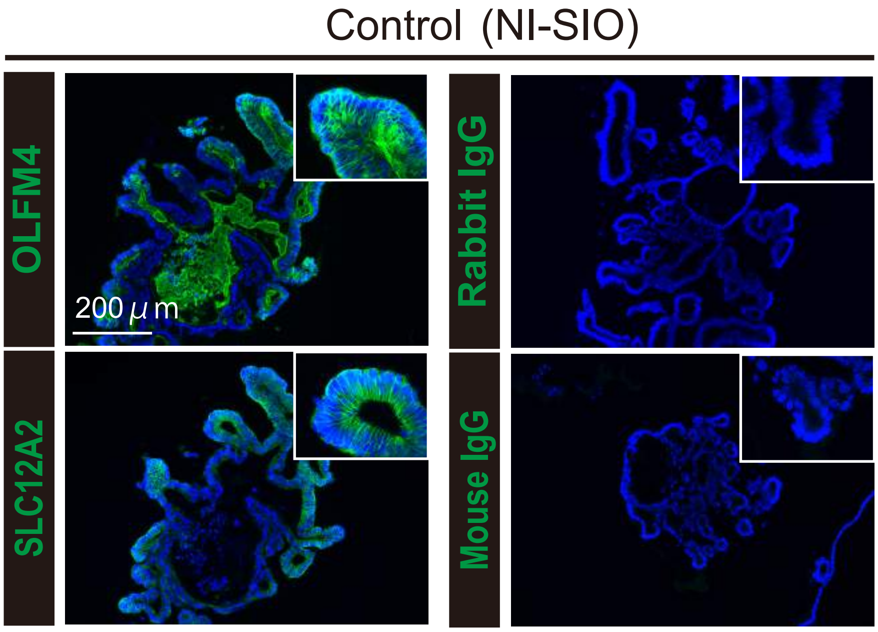

Supplementary Figure S3. Specificity confirmation for the immunostaing of OLFM4 and SLC12A2. Control organoids were stained by antibodies specific for OLFM4 or SLC12A2, or by their corresponding non-immunized control IgG. Positive signals are shown as green signals of the fluorescein-labeled tyramid. Note that positive signals of OLFM4 are observed in the lumens of the organoids, in addition to the epithelial cells of the budding region.
