# Supplementary material for: Single cell analysis of Crohn’s disease patient-derived small intestinal organoids reveals disease activity-dependent modification of stem cell properties
Source: J Gastroenterol. 2018 Jan 27;53(9):1035–47. doi: 10.1007/s00535-018-1437-3 (PMC6132922; doi:10.1007/s00535-018-1437-3)
Supplement: Supplementary file 4 — Supplementary material 4 (PDF 1218 kb) [file 535_2018_1437_MOESM4_ESM.pdf]

Supplementary Figure S4

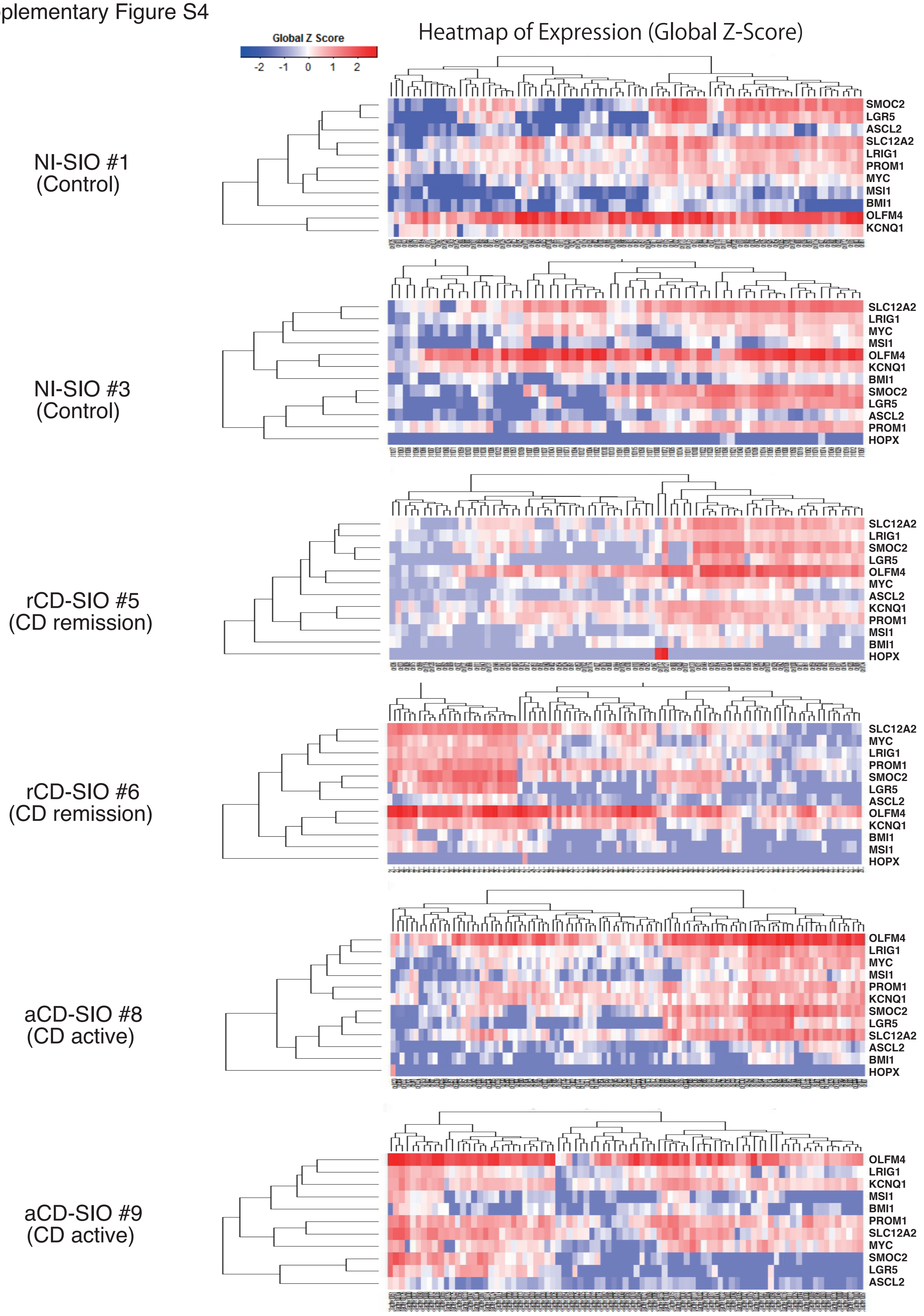

Supplementary Figure S4. Hierarchical analysis of the single-cell gene expression data acquired from patient-derived small intestinal organoids. Hierarchical analysis of the single-cell data was performed as described in Figure 3C. 135 cells were subjected to the analysis for each small intestinal organoid. Gene expression profiles of individual cells after elimination of outlier cells are shown.
