# Supplementary material for: Single cell analysis of Crohn’s disease patient-derived small intestinal organoids reveals disease activity-dependent modification of stem cell properties
Source: J Gastroenterol. 2018 Jan 27;53(9):1035–47. doi: 10.1007/s00535-018-1437-3 (PMC6132922; doi:10.1007/s00535-018-1437-3)
Supplement: Supplementary file 5 — Supplementary material 5 (PDF 234 kb) [file 535_2018_1437_MOESM5_ESM.pdf]

Supplementary Figure S5

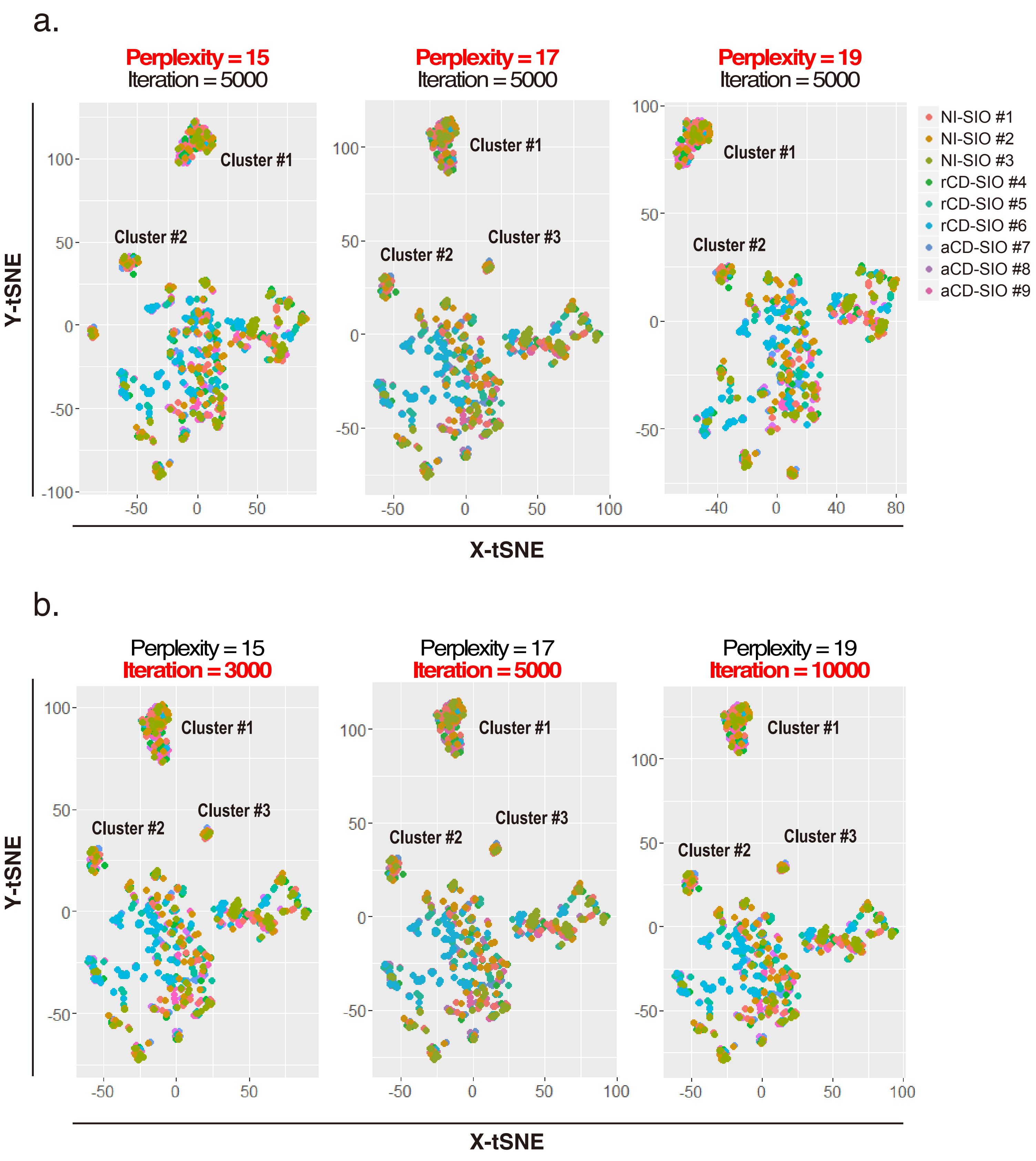

Supplementary Figure S5. Robustness of tSNE analysis.
tSNE analysis of dataset used in Figure 5A under different parameter conditions are shown. (a) Change in perplexity under fixed iteration number. (b) Change in iteration number under fixed perplexity.
