# Supplementary material for: Single cell analysis of Crohn’s disease patient-derived small intestinal organoids reveals disease activity-dependent modification of stem cell properties
Source: J Gastroenterol. 2018 Jan 27;53(9):1035–47. doi: 10.1007/s00535-018-1437-3 (PMC6132922; doi:10.1007/s00535-018-1437-3)
Supplement: Supplementary file 6 — Supplementary material 6 (PDF 228 kb) [file 535_2018_1437_MOESM6_ESM.pdf]

Supplementary Figure S6

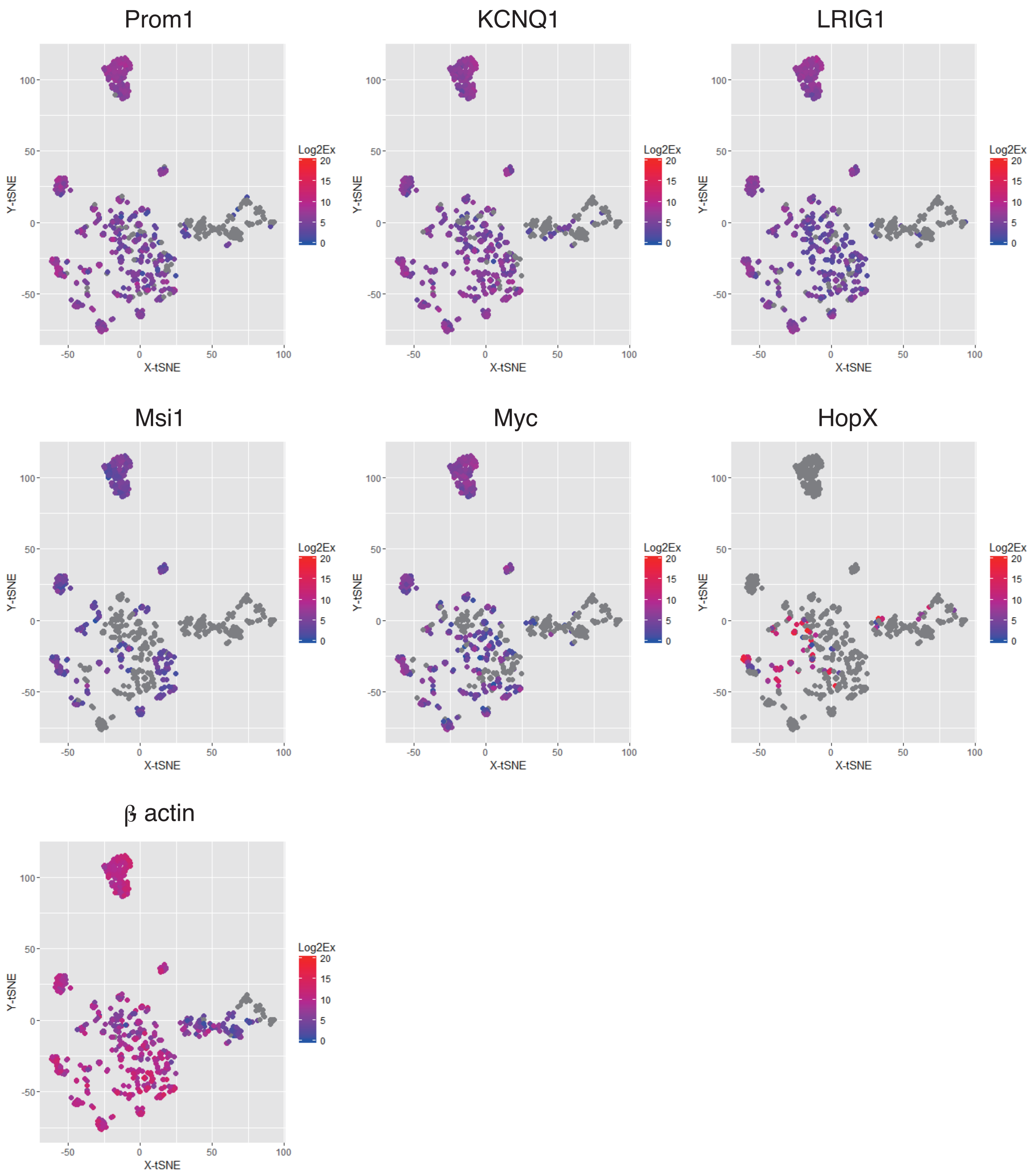

Supplementary Figure S6. Gene expression level of individual cells in clusters identified by tSNE analysis. Single-cell level expression of the designated genes are shown as an addtional data derived from tSNE analysis shown in Figure 5.
