# Supplementary material for: Single cell analysis of Crohn’s disease patient-derived small intestinal organoids reveals disease activity-dependent modification of stem cell properties
Source: J Gastroenterol. 2018 Jan 27;53(9):1035–47. doi: 10.1007/s00535-018-1437-3 (PMC6132922; doi:10.1007/s00535-018-1437-3)
Supplement: Supplementary file 7 — Supplementary material 7 (PDF 69 kb) [file 535_2018_1437_MOESM7_ESM.pdf]

Supplementary Table S1. Background descriptions of patients included in the study

ID	Organoid	Category	Age	Gender	Treatment	Sample location
#1	NI-SIO#1	Non-IBD	62	F	-	I00cm from ileocecal valve
#2	NI-SIO#2	Non-IBD	47	F	-	I00cm from ileocecal valve
#3	NI-SIO#3	Non-IBD	59	M	-	I00cm from ileocecal valve
#4	rCD-SIO#4	CD (remission)	39	M	5-ASA	I00cm from ileocecal valve
#5	rCD-SIO#5	CD (remission)	33	M	5-ASA + IFX Elementary Diet	I00cm from ileocecal valve
#6	rCD-SIO#6	CD (remission)	26	M	IFX+AZA	I00cm from ileocecal valve
#7	aCD-SIO#7	CD (active)	53	F	5-ASA + ADA	40cm from anastomotic site (right hemicolectomy)
#8	aCD-SIO#8	CD (active)	22	M	5-ASA	I00cm from ileocecal valve
#9	aCD-SIO#9	CD (active)	54	M	AZA	I00cm from ileocecal valve

Supplementary Table S2. List of genes used for the single cell analysis.

Gene	Probe set ID
GAPDH	Hs02758991 g1
ACTB	Hs01060665 g1
LGR5	Hs00969422 m1
OLFM4	Hs00197437 m1
ASCL2	Hs00944285 s1
BMI1	Hs00995536 m1
LRIG1	Hs00394267 m1
MSI1	Hs00159291 m1
HOPX	Hs04188695 m1
MYC	Hs00153408 m1
SMOC2	Hs01591663 m1
PROM1	Hs01009250 m1
SLC12A2	Hs00169032 m1
KCNQ1	Hs00923522 m1
